# Fertility preservation in young cancer patients

**DOI:** 10.4103/0974-1208.63113

**Published:** 2010

**Authors:** Ariel Revel, Shoshana Revel-Vilk

**Affiliations:** Department of Obstetrics and Gynecology, Hadassah Medical Center and Hebrew University-Hadassah Medical School, Jerusalem, Israel; 1 Department of Pediatric Hematology/Oncology, Hadassah Medical Center and Hebrew University-Hadassah Medical School, Jerusalem, Israel

**Keywords:** Cancer treatment, cryopreservation, fertility preservation

## Abstract

As a result of advances in treatment, almost 80% of children and adolescents who receive a diagnosis of cancer become long-term survivors. The increased survival rate of children and adolescents with cancer has resulted in a major interest in the long-term effects of cancer treatment on the possibility for future fertility. Currently established methods for the preservation of fertility are available only for pubertal males and females. Pubertal male cancer patients should be encouraged to freeze numerous sperm samples even when sperm count and motility are poor. In these cases, intracytoplasmic sperm injection is a powerful technique compared with intrauterine insemination since thawed sperm samples with poor parameters can produce relatively high fertilization rates resulting in normal pregnancies and deliveries. Married pubertal women should be proposed ovulation induction, follicular aspiration, and fertilization with husband sperm. Single women could benefit from vitrification of oocytes. This requires a delay of about 3 weeks in the commencement of chemotherapy to enable follicular growth. Fertility preservation for prepubertal patients is more of a problem. Young girls could be offered cryopreservation of gametes in the gonadal tissue. Cryopreservation of testicular tissue was suggested for fertility preservation for young boys, but this method is totally experimental and not currently offered. Discussing future fertility is part of the consultation of young female and male patients facing potentially gonadotoxic cancer therapy. It is the role of reproductive specialists to create various options in their laboratory to preserve fertility potential of cancer patients.

## INTRODUCTION

**Figure 1:**
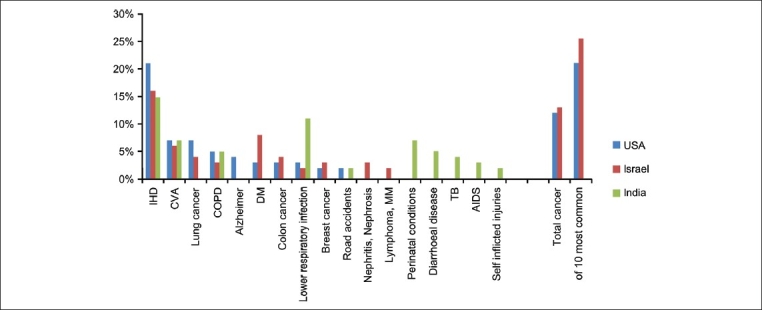
Causes of mortality between India, Israel, and USA. Whereas cancer is a common cause of death in the USA and Israel (12% and 13%, respectively), the situation in India is that it is not in the first ten common causes of death.

Causes of mortality differ between countries. Whereas cancer is a common cause of death in the USA and Israel (12% and 13%, respectively), the situation in India is different. Of the ten major causes of death, cancer is a cause of one out of 4 to 5 cases, in Israel and USA, respectively [Figure 1]. In India cancer is superceded by other causes of death and is not one of the top 10 causes of death.[1] However, with reduction in other causes of death, the World Health Organization (WHO) report that cancer has become India's leading cause of death.[2] Although cancer incidence has increased slightly over the past 30 years, mortality has declined dramatically for many cancers in this age group. The combined 5-year survival rate for all cancers in young age group has improved from less than 50% in the 1970s to 80% today, and the 10-year survival rate is almost 75%. Thus, as a result of advances in treatment, almost 80% of children and adolescents who receive a diagnosis of cancer become long-term survivors.

Cancer among children creates substantial public concern. In France 2000, new cases of cancer below the age of 15 are reported every year.[3] In the United States, each year approximately 150 out of every million children under 20 years of age are diagnosed with cancer.[4] Thus, about 12,400 children and adolescents in these countries alone are stricken. A male newborn has 0.32% probability of developing cancer by the age of 20 (i.e., 1 in 300 chance). In India, an estimated 1.5 million new cases of cancer are diagnosed annually. At any given time, there are about 2.5 million cases of cancer in India alone of which 500,000 are children.

The increased survival rate of children and adolescents with cancer has resulted in a major interest in the long-term effects of cancer treatment on the possibility of future fertility.

Germinal and stromal tumors of the ovary are common mostly in young women and children. Stage Ia dysgerminoma may be managed with unilateral adnexectomy and preservation of the uterus and contralateral adnexa. Early-stage epithelial ovarian cancer (stage Ia), which is less common in children and adolescents, may be managed with unilateral ovariectomy, which preserves the chance of natural pregnancy. The various options for fertility preservation currently available for females and males are summarized in Table 1.

**Table 1:** Options for fertility preservation

	Evidence	Delay for ovarian stimulation	Children born from procedure	Remarks
Females				
Embryo cryopreservation	Established	4-8 weeks	Millions	Sperm required
Oocyte cryopreservation	New	4-8 weeks	Hundreds	
Ovarian cortex cryopreservation and transplantation	Experimental	None	<10	
GnRH analogue ovarian protection	Controversial	None	?	
Males				
Ejaculate sperm cryopreservation	Established	-	Millions	
Alternative sperm cryopreservation	Established	-	Thousands	Testicular aspiration, extraction, electroejaculation
Testicular tissue cryopreservation	Experimental	-	None	

## OPTIONS FOR MALES

Sperm cryopreservation is the most established and effective method of fertility preservation in males.[5] Sperm is collected before initiation of cancer therapy. Later cryopreservation could result in risk that sperm DNA integrity or sample quality will be compromised. Underlying sperm quality may be poor for patients with certain cancer types, including testicular cancer, leukemia, and Hodgkin's.[6] Alternative methods of obtaining sperm besides masturbation include testicular aspiration or extraction, electroejaculation under sedation or anesthesia, or from a postmasturbation urine sample.[7]

During treatment, primordial sperm cells are susceptible to toxicity at all stages of life. Hormonal manipulations could theoretically protect testes from injury during cancer treatment. This is not, however, clinically available. With selected radiation fields, gonadal shielding can be applied during radiation therapy.[8] Testicular-tissue cryopreservation has resulted in only spermatogonia being detected.[9] *In vitro* maturation or germ-cell transplantation of testiclular cells or tissue may restore spermatogenesis in the future.

## OPTIONS FOR FEMALES

Embryo cryopreservation is an established technique with good pregnancy rates. Its use is, however, limited as it requires ovarian stimulation. It is thus appropriate only for adult female patients. Moreover, these women should be involved in a stable relationship. Additionally, the need for ovarian stimulation theoretically precludes this option for women with estrogen-sensitive tumors.

Collection of mature oocytes which requires ovarian stimulation, is appropriate only for adult patients.[10,11] Because of their large size, water content, and chromosomal architecture, mature female oocytes are extremely fragile. The spindle apparatus of the chromosome is easily damaged by intracellular ice formation during the freezing or thawing process.[12] Nevertheless, currently with a few hundred pregnancies from frozen or vitrified oocytes were reported.[13]

Ovarian-tissue cryopreservation, in which ovarian tissue is excised from the ovary and cryostored, is the only method that can be offered to prepubertal girls.[14–16] There are a large number of immature oocytes in the ovarian cortex at this age, when the primordial follicles contain prophase I oocytes. This technique has been accomplished in young children and the chance of later restoring fertility should be higher, theoretically, because the ovarian cortex contains an increased number of primordial and primary follicles in younger children.[10,15] Ideally, the stored ovarian tissue is thawed and autotransplanted into the donor once treatment has been completed.[17] To date, a dozen pregnancies have occurred in cancer survivors after autotransplantation of cryopreserved ovarian tissue. The last pregnancy reported being of twins.[18]

Concomitant treatment with gonadotropin-releasing hormone analogs (GnRHa) may prevent ovarian failure induced by cancer therapy.[19] These agents may protect against chemotherapy-induced follicular depletion, thus preserving primordial follicles. Although some studies have been performed in adult patients with cancer, these studies have not yet been extended to children. The effectiveness of this intervention is controversial.[20] Oophoropexy to divert the ovaries from the radiation field or gonad shielding is a potential strategy for preserving ovarian function during radiation therapy. Although ovarian transposition is relatively effective at preserving the endocrine function of the ovary, infertility is present in 85% of patients.[21]

## CASE SCENARIOS

This review will address the management of four common clinical situation encountered by fertility preservation specialists in the young age group.

### Case no. 1: A 23-year-old young woman with Hodgkin's lymphoma

Hodgkin's lymphoma is a malignant disease characterized by progressive enlargement of the lymph nodes, spleen, and general lymphoid tissue. Currently, the ABVD (adriamycin, bleomycin, vinblastine, and dacarbazine) chemotherapy regimen is the gold standard for treatment of Hodgkin's disease (HD). Developed in Italy in the 1970s, the ABVD treatment typically takes between 6 and 8 months, although longer treatments may be required. Although this regimen is considered not to be so toxic to ovarian function,[22] ABVD is not entirely without gonadotoxic effect.[23] Some Hodgkin's patients require more aggressive treatment such as the BEACOPP (doxorubicin, bleomycin, vincristine, cyclophosphamide, etoposide, prednisone) protocol either due to higher stage or relapse. This protocol is considerably more toxic to ovarian function.

It is important that this 23-year-old patient consults a specialist in reproductive medicine.

Regretfully, many hematologists do not refer patients to fertility consultants prior to chemotherapy. The aim of this consultation is to explain the effect of cancer treatment on future fertility for fertility preservation and to offer appropriate options. Since such young patients have an excellent future fertility prognosis following ABVD regimen, many physicians find it acceptable to offer no preservation. Young female patients are frequently offered GnRH analogue as a co-treatment that possibly reduces gonadotoxicity.[19] This approach is heavily criticized as lacking evidence-based proof of efficacy.[24] It should be stressed that ABVD could damage the primordial follicle pool and cause menopause and decreased fertility at a younger age. Moreover, if fertility preservation method is considered, the best time is before the patient receives any chemotherapy. Should this 23-year-old HD patient request fertility preservation, the best approach would be to offer cryopreservation of gametes. If she is married, we would propose ovulation induction followed by follicular aspiration and fertilization with the husband's sperm. Frozen embryos were shown to be able to obtain pregnancies even years following their cryopreservation.[25] Millions of children worldwide were born following *in vitro* fertilization (IVF), many of them following frozen embryo transfer. Pregnancy rate above 35% per transfer is expected for these patients. Preservation of 10 frozen embryos could suffice to ensure future live birth.

Single women could benefit from vitrification of oocytes.[26,27] Similar to IVF, this requires a delay of about 3 weeks in commencement of chemotherapy to enable follicular growth and follicular aspiration. Results following thawing and fertilization are increasing. A few hundred children world wide were born following cryopreservation of unfertilized oocytes. It is estimated that 15-20 oocytes should be vitrified to ensure future child birth at this age. Follow-up of the children born following this technique appears to be normal.

### Case no. 2: A 17-year-old male adolescent with acute lymphoblastic leukemia

Of children with acute lymphoblastic leukemia (ALL), 20% exhibit high-risk characteristics. In addition, 20% of the patients at standard risk experience relapse that requires intensive therapy with alkylating agents, hematopoietic stem cell transplantation, or testicular irradiation. These patients have high risk for long-term testicular dysfunction. The number of germ cells expressing spermatogonial markers in boys with ALL is significantly correlated to the cyclophosphamide therapy cumulative dosage and individual sensitivity.[28]

UK and American Society of Clinical Oncology (ASCO) guidelines recommend that the implications of oncological treatment for fertility are discussed with all patients. Although all hematooncologists recognize that semen cryopreservation helps the patient psychologically, many do not refer young men and adolescents as they believe that semen freezing delays cancer treatment. Moreover, physicians face the difficult task of balancing between their ideas of what is in the best interest of the adolescent and accommodating parental wishes. Semen cryopreservation should sometimes be discussed separately with adolescent and parents.[29]

Male cancer patients should be encouraged to freeze numerous sperm samples even when sperm count and motility are poor. In these cases, intracytoplasmic sperm injection (ICSI) is a powerful technique compared with intrauterine insemination (IUI) since thawed sperm samples with poor parameters can produce relatively high fertilization rates resulting in normal pregnancies and deliveries. The possibility to repeat fertility treatments even in the face of a limited number of sperm samples appears to be of importance,[5,30] emphasizing the need for multiple sperm samples.

### Case no. 3: A 12-year-old girl with Ewing sarcoma

Ewing sarcoma is a malignant primary bone tumor common in adolescents and young adults. Because almost all patients with apparently localized disease at diagnosis have occult metastatic disease, multidrug chemotherapy including gonadotoxic ifosfamide and etoposide, as well as local disease control with surgery and/or radiation are indicated.[31] At this age, cryopreservation of gonadal tissue should be considered. Although this procedure is still considered experimental, currently this is the only option for fertility preservation in a young girl.

To minimize the risk of an additional anesthesia, cryopreservation of ovarian tissue could be performed in combination with another surgical procedure such as the insertion of a central venous catheter. Using this approach in young girls was shown to be safe.[15] The lowest age at which this approach is recommended is not defined. Based on multidisciplinary discussions, we have recommended that ovarian tissue cryopreservation should be offered from 3 years of age.[16] Moreover, oocytes could be aspirated, matured, and cryopreserved even in premenarcheal patients.[15]

To date, transplantation of frozen-thawed ovarian tissue has yielded 11 live births worldwide. All these pregnancies were obtained after orthotopic transplantation, either after natural conception[32–34] or IVF.[23,35]

The possibilities to cryopreserve ovarian tissue exist. The first, development by Gosden in the sheep model involves cutting the ovarian cortex into tiny pieces, which are the cryopreserval either by slow freeze or more recently, by vitrification. All pregnancies and deliveries were obtained by this method. Ischemic damage to the ovarian cortex, which occurs mostly postgrafting until neovascularization resumes, decreased the follicle pool.

Nevertheless such grafts appear to function for up to 3-5 years and enable resumption of ovarian function. In order to circumvent this ischemia, we[36] and others[37] have proposed to freeze an intact ovary with its vascular pedicle. Although an interesting concept, this approach is technically very difficult mainly due to the need to reanastomoze very small diameter arteries and veins and the lack of efficient cryopreservation methods of organs.

### Case no. 4: A 7-year-old boy with brain tumor

Modern therapy for children with central nervous system (CNS) malignancies often includes both surgical resection and a combination of CNS-directed radiation therapy (RT) and chemotherapy. This multimodal approach enables 5-year survival of 3 out of 4 boys younger than 20 years who are diagnosed with a CNS malignancy. Exposure to multimodal therapy may, however, increase risk of long-term morbidity.[38] Fertility in adult life may be impaired by gonadotoxic therapies. Impaired spermatogenesis has been observed in brain tumor survivors secondary to chemotherapy and in combination with spinal irradiation.[39] Inhibin B and FSH levels can assist in evaluating the gonadal damage.[39] Cases of panhypopituitarism secondary to treatment of brain tumors can be treated by gonadotropin replacement therapy, which could restore normal spermatogenesis.[40] Increased awareness of hemato-oncologists and other physicians treating male cancer patients to advances in reproductive techniques is of high priority. Obtaining gametes from prepubertal boys is a complex task, which requires cryopreservation of testicular tissue.[41] For young boys who do not yet produce spermatozoa, cryopreservation of immature testicular tissue is an option to preserve their fertility, albeit still experimental.

Some centers have recently started to offer the cryopreservation of testicular tissue, though no transportation has been performed in human. Frozen testicular tissue could be thawed when the childhood cancer survivor reaches adulthood and it is found to be azoospermic. Existing approach to immature testicular tissue cryopreservation and fertility restoration are currently being investigated and current knowledge on *in vitro* spermatogonial stem cell (SSC) protection has been published.

## CONCLUSION

Cancer detection and treatment follows international protocols and guidelines. This has enabled to increase survival and decrease adverse effects. Reduced fertility is a common long-term effect of cancer therapy. For young people and their parents, this is a major cause of concern when dealing with the diagnosis and future treatment of their disease. Assisted reproductive technology currently offers a variety of fertility preservation techniques. These possibilities are available in most countries. Although costly, these methods are increasingly in demand. We believe that discussing future fertility is part of the consultation of young female and male patients facing potentially gonadotoxic cancer therapy. Oncohematologists in pediatrics and young adults should recognize the various options available in their country. Cross-border reproductive tourism is however in demand, when options are not available in the patient's homeland. It is the role of reproductive specialists to create various options in their laboratory to preserve fertility potential of cancer patients.
